# Relative intensities and compositions of multifactorial peak kinematic and mechanical demands in elite youth soccer

**DOI:** 10.3389/fspor.2025.1616921

**Published:** 2025-09-04

**Authors:** Farzad Yousefian, Abdullah Zafar, Fábio Y. Nakamura, Pedro Menezes, João Brito, Bruno Travassos

**Affiliations:** ^1^ Research Center in Sports Sciences, Health Sciences and Human Development (CIDESD), Department of Sports Sciences, University of Beira Interior, Covilhã, Portugal; ^2^ Portugal Football School, Portuguese Football Federation, Oeiras, Portugal; ^3^ Department of Biomedical Sciences, University of Montreal, Montreal, QC, Canada; ^4^ Research Center in Sports Sciences, Health Sciences and Human Development (CIDESD), University of Maia, Maia, Portugal; ^5^ Clube de Regatas do Flamengo, Rio de Janeiro, Brazil; ^6^ CIPER, Faculdade de Motricidade Humana, Universidade de Lisboa, Lisbon, Portugal

**Keywords:** football, match analysis, peak periods, GPS, youth tournament

## Abstract

**Background:**

Characterizing the most demanding passages (MDP) of physical activity during soccer competition is essential for optimizing training prescription and player monitoring. However, research investigating kinematic and mechanical MDP using a multifactorial criterion variable approach in elite youth soccer players remains limited. This study examined the relative intensities and compositional structure of multifactorial kinematic and mechanical MDP across different durations in an elite youth international tournament.

**Methods:**

Locomotor activity data were collected from 17 elite youth players across five matches of an international tournament using GPS technology. Kinematic and mechanical MDP were identified using multifactorial criterion variables: MDPk (kinematic) and MDPm (mechanical). Linear mixed models assessed relative intensities (m·min^−1^ or efforts·min^−1^) and the univariate constituent compositions of MDPk [moderate-speed running [MSR], high-speed running [HSR], sprinting [SPR]] and MDPm [high-intensity accelerations [ACC3], decelerations [DEC3]] across rolling (R’) 1-, 3-, and 5-minute durations, comparing matches and positional groups (central, lateral).

**Results:**

Differences between matches and positions were observed only for R1’ MDPk intensities. Both MDPk and MDPm intensities decreased as duration increased. Between-match differences were observed for MDPk composition, particularly for %HSR and %SPR. MDPk composition was duration-dependent, as %HSR and %SPR were greater in R1’ than R3’ and R5’, while %MSR followed the opposite trend. In contrast, MDPm composition remained stable across durations.

**Conclusions:**

Practitioners should consider both the relative intensities and compositional structures of MDPk and MDPm when evaluating multifactorial peak demands in soccer. Analyzing kinematic and mechanical MDP as multifactorial constructs offers critical insights into the contribution of specific locomotor demands across various durations. This approach emphasizes the importance of duration-specific analyses in optimizing training, recovery, and match preparation strategies, thereby facilitating targeted training interventions and enhancing player readiness for competition.

## Introduction

Characterizing the locomotor demands of soccer match play is essential for designing effective training programs that prepare youth players for competition and support their long-term development (1). However, informing training prescriptions using total or average match demands may inadequately prepare players for peak external loads, known as the most demanding passages (MDP) during the match, which may lead to psychophysiological maladaptation and compromised performance (2, 3). The MDP, also referred to as the “most intense periods” (4), “peak match demands” (5), and “worst-case scenarios” (6) represents the period of maximal physical activity during the match (7, 8). Consequently, understanding MDP demands in professional youth competition is important for optimizing player development and facilitating their progression to senior level competition (5, 9).

Previous research in professional youth soccer has examined the MDP of various kinematic (e.g., running distance) and mechanical (e.g., accelerations and decelerations) performance variables across different rolling average durations (10, 11). An established inverse relationship exists between intensity and duration, whereby shorter MDP durations (e.g., 1 min) elicit the highest relative intensities compared to longer durations (e.g., 3 and 5 minutes) (4, 10, 12). This trend suggests that as MDP duration increases, the intensity of performance variables decreases. Such reductions in intensity over longer durations may be influenced by multiple technical/tactical, physiological, and match-specific contextual factors. For instance, match stoppages and changes in ball status, transitions between tactical phases and ball possession status, and shifts in the energy system contributions may all contribute to the attenuation of MDP intensities over extended durations (13–15). Consequently, shorter-duration MDP may better capture short periods of peak demands that define critical match moments, while longer-duration MDP might include lower-intensity phases that attenuate overall intensity measures. However, there is limited information regarding the selection of MDP durations and their implications for interpreting results and informing training prescription (4, 16). Additionally, kinematic and mechanical MDP demands have been reported to be influenced by player positions, with central positions (e.g., central defenders and midfielders) typically exhibiting lower demands than lateral positions (e.g., wide defenders and midfielders) (4, 16, 17).

Despite the insights gained from previous research, the predominant reliance on discrete (univariate) performance variables to identify and characterize MDP match demands may be reductionist, limiting the applicability of findings in practice (7, 8, 18). For example, while previous studies have reported the MDP for distances covered at speed thresholds ≥21 km·h^−1^ (TD21) (7), ≥19.8 km·h^−1^ (high-speed running, HSR) (18), and ≥24 km·h^−1^ (sprinting, SPR) (8), these investigations did not account for the absolute or relative contributions of additional locomotor activities during these periods (12, 19). Given the multifaceted and complex nature of soccer performance, where players frequently engage in dynamic transitions across multiple kinematic and mechanical locomotor intensities throughout the match, considering the MDP as a multifactorial construct may increase the specificity of training by enhancing the transferability of knowledge from monitoring to training design (16, 17). In effect, such an approach may facilitate more effective evaluations of match and training demands, providing a more comprehensive understanding of players’ activity profiles, and ultimately improving long-term player development strategies (17, 19).

Recent research analyzed the kinematic and mechanical MDP using respective multifactorial criterion variables MDPk and MDPm across 5-minute periods in professional male soccer players (17, 19). The authors reported that the univariate MDP of the respective constituent variables of MDPk (MSR, HSR, and SPR) and MDPm (ACC3 and DEC3) are comparable in both magnitude and frequency distribution across each half (19). Furthermore, MDPk and MDPm demands are position-specific, with lateral positions (full-backs and wide midfielders) showing higher kinematic and mechanical demands than central positions (central defenders and midfielders), which also exhibit lower values in both MDPk and MDPm intensities and compositions (17). The studies highlight the importance of using a multifactorial approach to provide greater ecological validity through integrating the relative intensities and compositions of MDPk and MDPm. Such methods enhance our understanding of how different positional roles experience and respond to peak physical demands, allowing for nuanced interpretations of fatigue accumulation and recovery needs based on changes in MDP intensity and composition (12, 15, 19). However, it remains unclear as to whether similar results apply across different durations or in youth soccer. The influence of additional duration periods on the constituent composition of multifactorial MDP performance variables also warrants further exploration.

In elite youth soccer, tournaments often consist of congested fixture periods, with multiple matches played over short timeframes (e.g., three matches within eight days), resulting in limited recovery (≤72 hours) between consecutive matches (9, 20, 21). Such conditions can exacerbate post-match psychophysiological stress, thereby increasing the risk of impaired performance and injury (20, 21). However, research in youth soccer has reported no significant differences in high-intensity kinematic and mechanical MDP performance between successive matches or between congested and non-congested periods (7, 8, 18). For example, Jimenez et al. (2023) reported no differences in high-intensity kinematic and mechanical MDP across 1-, 5-, and 10-min periods in elite U19 players when comparing congested and non-congested fixtures. Similarly, Castellano et al. (2019) observed no changes in distances covered ≥21 km·hr^−1^ MDP across 1-, 3-, 5-, or 10-min periods when three matches were played within one week in elite U19 players. Doncaster et al., (18) likewise reported no differences in HSR (≥19.8 km/hr) MDP across 1-, 3-, and 5-min periods between one-match and two-match microcycles in elite U23 players. Collectively, these findings suggest that players can maintain MDP performance during congested match fixtures; however, no studies have specifically examined MDP demands in elite youth players during an international tournament.

Taken together, a critical gap remains in the literature on composite MDP demands, particularly within elite youth soccer and, more specifically, within international tournament contexts. Therefore, this study aimed to investigate the relative intensities and compositions of multifactorial kinematic (MDPk) and mechanical (MDPm) MDP profiles during an elite youth international tournament across multiple rolling average durations according to matches and playing positions.

## Methods

### Subjects

Data were collected from 17 elite male players [age: 18.5 ± 0.9 y (range: 17–20 years); height: 180.4 ± 5.0 cm; weight: 72.5 ± 3.5 kg] belonging to the 2024 CONMEBOL Under-20 (U20) Copa Libertadores winning team. This study was approved by the university ethics committee (ERB#: 210/2024) and adhered to the principles of the Declaration of Helsinki.

### Methodology

Player locomotor activities were collected across all matches of the elite international youth tournament using 10-Hz global positioning system (GPS) devices integrated with micro-electromechanical system (MEMS) and Global Navigation Satellite System (GNSS) (Catapult Vector S7, Catapult Sports, Melbourne, Australia). The tracking devices demonstrate valid and reliable measurements of speed, distance and sprint accelerations [coefficients of variation (CV) = ≤2%, ICC = 0.84 −0.99] and have been previously used in senior and elite youth soccer studies (13, 22–24). Overall, 10 Hz GPS devices reliably quantify accelerations (CV = 1.9%–4.3%) but show variable reliability for decelerations (CV = 2.5–10.9%) and consistently measure distance and multidirectional activities with acceptable accuracy (CV = 2.0%–5.3%) (11, 25–27). The tournament consisted of five official matches, including three group-phase matches and two knockout-phase matches (semi-final and final matches). Only outfield players who started and completed a minimum of 75 minutes per match were included in the analysis, and goalkeepers were excluded, resulting in a total of 46 match observations (2.6 ± 1.5 per player; range 1–5). The observed team won all five matches played across 13 days and consistently played in a 1-4-3-3 formation. Players were initially categorized by position as follows: central defender (CD; *n* = 10 observations), full-back (FB; *n* = 7), central midfielder (CM; *n* = 14), winger (WG; *n* = 10), and forwards (FW; *n* = 5). Subsequently, players were analyzed as central (CEN; CD, CM, and FW) and lateral (LAT; FB and WG) positions.

Each player wore the same GPS unit throughout the tournament, placed in a fitted vest between the scapulae. After each match, raw velocity data (0.1-s intervals) were downloaded using proprietary software (Openfield, Catapult Innovations, Melbourne, Australia), transferred to a spreadsheet, and analyzed using Python programming software (version 3.9). A rolling average analysis was conducted across 1-, 3-, and 5-minute durations and the maximal value for each performance variable was designated as the MDP. Kinematic and mechanical MDP were identified using composite criterion variables: MDPk and MDPm. MDPk consisted of the maximal total distance covered across moderate-speed running (MSR; 15–19.8 km·hr^−1^), high-speed running (HSR; 19.8–25.2 km·hr^−1^), and sprinting (>25.2 km·hr^−1^) thresholds, while MDPm comprised the maximal sum of high-intensity acceleration (ACC3; ≥3 m·s^−2^) and deceleration (DEC3; ≤−3 m·s^−2^) efforts for each duration (17, 19). Relative MDPk (m·min^−1^) and MDPm (n·min^−1^) intensities were calculated to compare across rolling average durations. The compositions of MDP criterion variables were defined as the percentage of each constituent variable within the respective composite criterion variable.

### Statistical analysis

Linear mixed models (LMM) were used to assess differences and interactions between fixed effects, including durations (R1’, R3’, and R5’), matches (M1-5), and positions (central and lateral), with player and match identifiers included as random effects. Dependent variables included MDPk and MDPm relative intensities (in meters or number of efforts per minute, respectively) and the relative composition of their respective constituent variables (% of MDPk and % of MDPm). Normality of residuals was assessed using the Shapiro–Wilk test and QQ-plot visual inspection. Non-normally distributed data were log-transformed and subsequently back-transformed, with results presented as mean ± SD. For significant results, Bonferroni *post-hoc* tests were conducted for pairwise comparisons. Effect size was calculated using Cohen's *d* and interpreted as: 0.2–0.6 (small), 0.6–1.2 (moderate), 1.2–2.0 (large), and ≥2.0 (very large). All statistical analyses were performed using IBM SPSS version 27 (IBM Corp., Armonk, NY, USA). Significance was set at *p* ≤ 0.05, with data presented as mean ± SD.

## Results

### Match & position intensities

**Figure 1:**
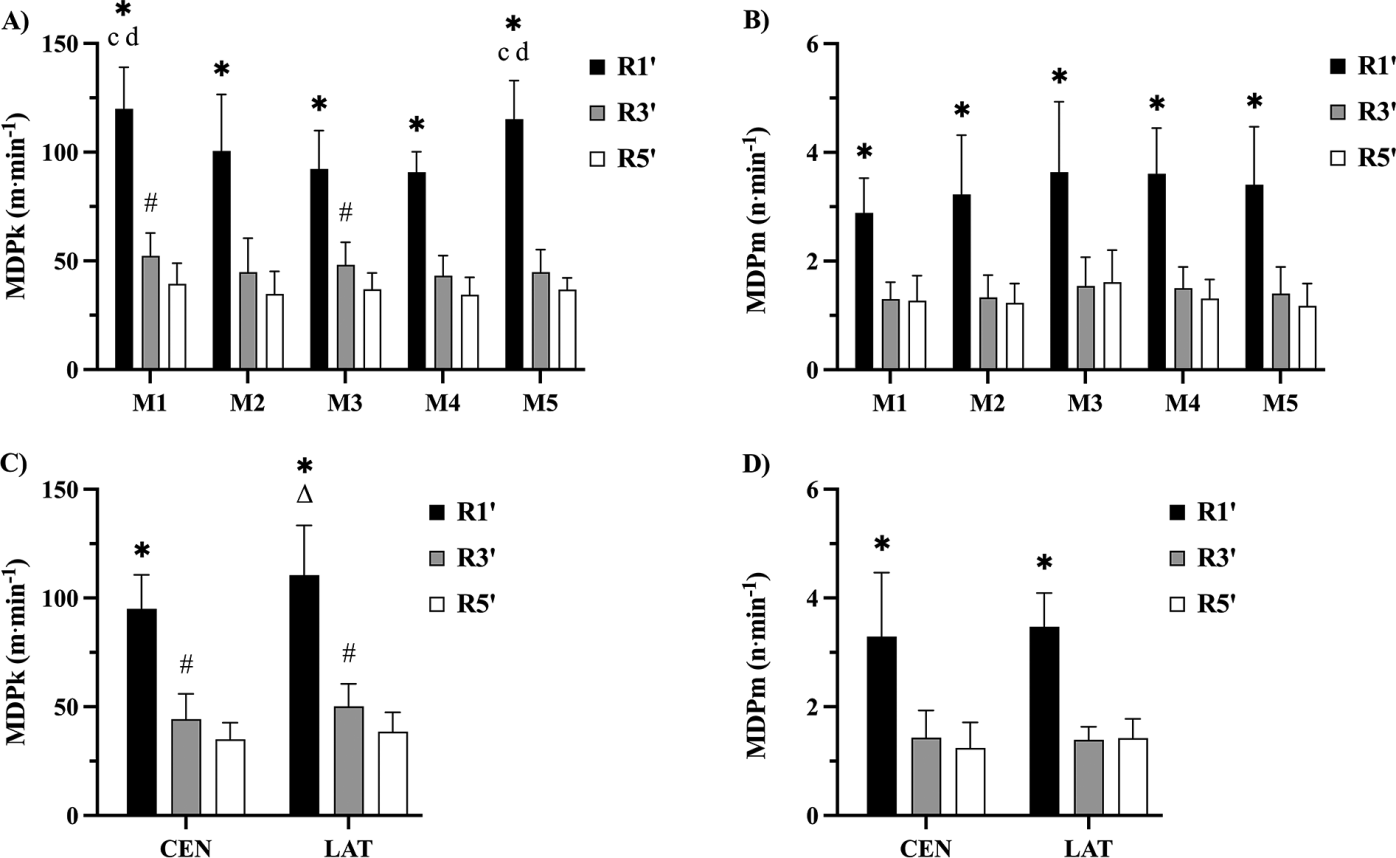
Relative MDPk and MDPm intensities across matches **(A,B)** and positions **(C,D)** according to rolling average duration. Statistical differences (*p* ≤ 0.05): c > M3; d > M4; * > R3’ & R5’; # > R5’; Δ > CEN. M, match; R’, rolling average duration (min); CEN, central positions; LAT, lateral positions.

Figure 1 displays the relative MDPk and MDPm intensities across matches and positions according to duration. Significant interactions between duration and matches revealed differences for R1’ MDPk intensities between matches (*p* ≤ 0.01; *d* = 1.2–2.0). Across all matches, R1’ MDPk intensities were higher than R3’ and R5’ (*p* ≤ 0.001; *d* = 2.5–6.2), while R3’ was higher than R5’ in matches 1 and 3 (*p* ≤ 0.05; *d* = 1.2). No significant differences in MDPm intensities were observed between matches; however, MDPm R1’ intensities were higher than R3’ and R5’ across all matches (*p* ≤ 0.001; *d* = 2.5–2.6; Figures 1A,B).

Duration and position interactions showed moderately higher R1’ MDPk intensities for lateral compared to central positions (*p* ≤ 0.001; *d* = 0.8). Within each position, R1’ intensities were higher than R3’ and R5’ (*p* ≤ 0.001; *d* = 3.4–5.0), and R3’ was higher than R5’ (*p* ≤ 0.05; *d* = 0.9–1.2). No significant differences were observed for MDPm intensities between positions; however, R1’ MDPm intensities were higher than R3’ and R5’ (*p* ≤ 0.001; *d* = 2.5–2.6; Figures 1C,D).

Overall MDPk and MDPm intensities across durations were R1’: 102.6 ± 21.1 m·min^−1^; R3’: 46.5 ± 11.4 m·min^−1^; R5’: 36.4 ± 8.1 m·min^−1^; and R1’: 3.4 ± 1.0 n·min^−1^; R3’: 1.4 ± 0.4 n·min^−1^; R5’: 1.3 ± 0.4 n·min^−1^, respectively.

### Match & position compositions

**Figure 2:**
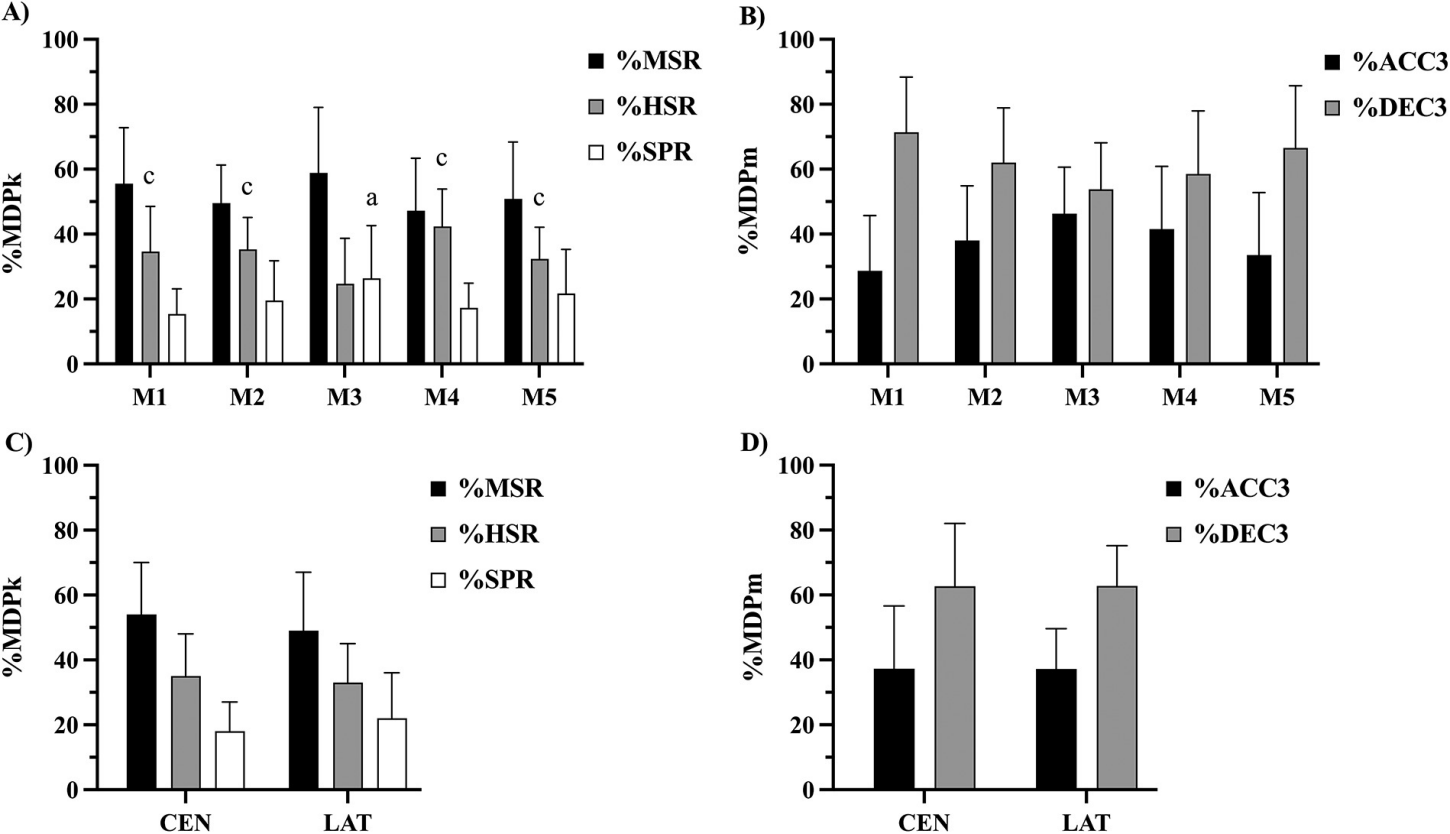
Relative MDPk and MDPm compositions across matches **(A,B)** and posistions **(C,D)** according to their respective constituent variables, averaged across all rolling average durations. Statistical differences (*p* ≤ 0.05): a > M1; c > M3. M, match; CEN, central positions; LAT, lateral positions; MSR, moderate-speed running; HSR, high-speed running; SPR, sprinting; ACC3, high-intensity acceleration; DEC3, high-intensity deceleration.

Figure 2 displays the mean relative constituent variable compositions of MDPk and MDPm across all rolling average durations, according to matches and positions. Statistically significant main effects for matches were observed for all constituent variables of MDPk (%MSR, %HSR, and %SPR) and MDPm (%ACC3 and %DEC3). However, *post hoc* pairwise comparisons with Bonferroni correction revealed no significant differences between individual matches for %MSR, %ACC3, and %DEC3. Moderate to large differences between matches were found for %HSR (*p* ≤ 0.05; *d* = 0.7–1.4) and %SPR (*p* ≤ 0.05; *d* = 0.9; Figures 2A,B). No statistically significant differences were observed for MDPk and MDPm compositions between positions (Figures 2C,D).

**Figure 3:**
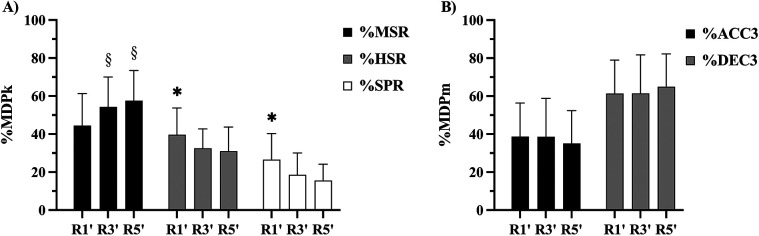
Relative MDPk **(A)** and MDPm **(B)** compositions according to rolling average duration. Statistical differences (*p* ≤ 0.05); § > R1’; * > R3’ & R5’. R’, rolling average duration (min); MSR, moderate-speed running; HSR, high-speed running; SPR, sprinting; ACC3, high-intensity acceleration; DEC3, high-intensity deceleration.

Figure 3 displays the relative constituent variable compositions of MDPk and MDPm according to duration. Irrespective of match and position, the rolling average duration differentially influenced the relative constituent variable compositions of MDPk. The %MSR was significantly lower in R1’ (∼44%) compared to R3’ (∼54%) and R5’ (%58%; *p* ≤ 0.001; *d* = 0.6–0.8), while %HSR (R1’: ∼40%; R3’: ∼33%; R5’: ∼31%) and %SPR (R1’: ∼27%; R3’: ∼19%; R5’: ∼16%) revealed the opposite trend (*p* ≤ 0.001; *d* = 0.6–1.0). In contrast, no significant differences were observed in MDPm composition across durations for %ACC3 (∼40%) and %DEC3 (∼60%).

## Discussion

This study investigated the relative intensities and constituent compositions of the most demanding passages of kinematic (MDPk) and mechanical (MDPm) activities across matches, positions, and rolling average durations during an elite youth (U20) international tournament. The key findings indicate that (i) despite fluctuations in MDPk and MDPm intensities across matches and positions, significant differences were observed only for short-duration (R1’) MDPk intensities; (ii) relative MDPk and MDPm intensities decreased with increasing duration; and (iii) significant fluctuations in MDPk compositions across matches were associated with specific constituent variables, particularly %HSR and %SPR. These findings underscore the multifactorial nature of MDP demands during match play and emphasize the importance of integrating duration-specific analyses for training and match preparation.

The rolling average duration and MDP criterion variables influenced differences in match and positional MDP demands. Significant match-specific differences were observed only in R1’ MDPk intensities, suggesting that peak kinematic stressors fluctuate across matches in elite male youth competition. In contrast, the relatively stable MDPm intensities imply greater consistency in neuromechanical load, reinforcing the notion that high-intensity kinematic workload is more susceptible to match-specific factors such as tactical strategies, opposition quality, and situational game demands (9, 13, 19). As the duration increases, tactical adjustments, increased stoppages, and fluctuations in match tempo may reduce the overall magnitude of MDP performance (6, 13, 14). The absence of significant differences in longer-duration MDPk suggests that these fluctuations primarily affect transient peak intensities rather than more sustained running outputs (8, 18, 28). Additionally, transitions between anaerobic and aerobic energy systems over prolonged periods may further limit players’ abilities to sustain high-intensity activities, which may further influence MDP demands (15, 29). These findings highlight the importance of match-specific analyses to contextualize peak workload demands.

The tournament format presents a unique case study for analyzing MDP performance during congested fixture periods. In line with previous research in U19 and U23 players, despite fluctuations between matches, MDP demands remained stable throughout the tournament, suggesting that elite youth players can maintain peak performance across successive matches within congested fixtures (7, 8, 18). Effective team management strategies, such as limiting player exposure between successive matches and implementing recovery interventions, which may have contributed to mitigating psychophysiological stress, as suggested by these findings (9, 18, 20, 21). Future research should investigate these potential factors by incorporating additional teams, tournaments, and match contexts to gain a more comprehensive understanding of such effects.

A notable positional difference was observed in R1’ MDPk intensities, where lateral positions exhibited significantly higher R1’ MDPk (110.5 ± 22.9 m·min^−1^) compared to central positions (95.0 ± 15.7 m·min^−1^). This finding is consistent with previous research demonstrating that wide players, particularly full-backs and wingers, experience greater sprinting and high-speed running demands due to their tactical roles and involvement during attacking and defensive phases (11, 12, 15). However, no significant positional differences were found for MDPm intensities, suggesting that while lateral players perform more high-speed locomotor activities, the frequency of high-intensity mechanical efforts is comparable across positions (30). Future research should refine positional classifications to more clearly differentiate the specific roles of central defenders, midfielders, and forwards, thereby optimizing training prescriptions based on position-specific MDP demands. For example, grouping central defenders, central midfielders, and forwards likely reduced the contrast with lateral positions, given the distinct MDP demands previously reported across these roles. For instance, in professional senior players, the peak 3-min HSR was greater in lateral positions (FB and WM) than in other roles, while FB and FW exhibited higher peak 1-min and 5-min SPR compared to MF (16).

Between-match differences were observed for MDPk compositions, particularly in %HSR and %SPR. The moderate to large differences in these MDPk constituent variables highlight the fluctuating nature of MDP demands (9, 13, 14). For instance, the lowest %HSR and highest %SPR observed in Match 3 may indicate a greater reliance on explosive efforts in that match, potentially reflecting tactical shifts or opponent-specific adaptations (9). These findings could indicate a match characterized by frequent counterattacking situations or high defensive compactness, requiring players to perform more maximal sprinting actions within the MDPk (13, 14). However, the lack of significant differences in %MSR, %ACC3, and %DEC3 between matches suggests that specific kinematic and mechanical demands remain relatively stable, likely due to the inherent consistency of mechanical actions in soccer, where repeated accelerations and decelerations occur independently of match-specific running patterns (31). These findings highlight the importance of match-specific monitoring of the intensities and compositions of multifactorial MDP, as individual match contexts may impact the intensity and distribution of high-speed activities, requiring adaptive training and recovery protocols.

Previous research in senior players reported R5’ MDPk compositions (∼55% MSR, ∼30% HSR, and ∼15% SPR) comparable with those observed in this study with youth players (∼55% MSR, ∼30% HSR, and ∼20% SPR) (19). These findings suggest that the relationship between duration and MDPk composition may be comparable across cohorts, despite differences in relative intensities (senior: ∼46 m·min^−1^; youth: ∼36 m·min^−1^). Further research across larger samples and diverse cohorts is necessary to establish differences in MDPk and MDPm according to performance levels (5). In contrast, MDPm compositions between cohorts were similar, with %ACC3 and %DEC3 comprising ∼40% and ∼60% of MDPm, respectively (17).

While MDPk and MDPm compositions did not differ significantly by positions, the observed trends were consistent with prior studies on position-specific MDP demands (4, 12). Lateral positions (FB, WG) exhibited lower %MSR, similar %HSR, and higher %SPR compared to central positions (CD, CM, FW), although these differences were not statistically significant. The limited sample size and positional categorization in this study may have influenced these findings. Prior studies in Swedish first-division male players reported significant statistical differences in MDPk between central (CD, CM, FW) and lateral positions (FB, WM) (17). MDPm compositions, however, were comparable across positions, aligning with our findings. Further research with larger samples and refined positional classifications could reveal additional insight surrounding variations in MDP demands, allowing for tailored training interventions that target specific locomotor profiles. For example, central defenders may benefit from training focused on moderate-speed running endurance, while wide midfielders and forwards may require more sprint-based conditioning (32, 33).

MDPk composition was significantly influenced by duration, with shorter durations (R1’) characterized by higher %HSR (∼40%) and %SPR (∼27%), whereas longer durations (R5’) exhibited reductions in these variables (%HSR: ∼31%; %SPR: ∼16%) and increased %MSR (∼58%). These findings suggest a progressive shift from high-intensity running to moderate-speed activity as duration increases (4, 15). While the inverse relationship between MDP intensity and duration is well established, this study is the first to report changes in MDPk composition according to rolling average duration. Despite similar MDPk intensities between players or matches, variations in constituent variable compositions (%MSR, %HSR, %SPR) may impose different metabolic demands, shifting between aerobic and anaerobic energy pathways (15, 29). Consequently, variations in MDPk composition may also contribute to transient fatigue post-MDP, whereby decrements in high-intensity activities are observed following short (R1’) compared to longer durations (R3’ and R5’) (28). Such shifts may reflect match-specific tactical and situational contexts (13, 14). For instance, Bortnik et al. (13) reported that attacking and defensive transitions require higher sprinting and high-speed running and lower acceleration/deceleration loads across tactical transitions lasting <12 s. Thus, the relationship between MDP composition and tactical variability requires further study, as tailored interventions according to MDP duration and composition may enhance preparation for both high-intensity sprinting and sustained moderate-speed efforts (32, 33). High-intensity interval training (HIIT) and speed endurance training can enhance %SPR capacity, improving the ability to sustain high-intensity efforts and effective recovery (9, 32, 34).

Interestingly, MDPm compositions remained stable across durations, with %ACC3 (∼40%) and %DEC3 (∼60%) consistently observed. These results align with previous research reporting comparable values across R5’ periods (17), suggesting that mechanical loads (acceleration and deceleration) are relatively constant regardless of MDP duration. The relative stability of mechanical demands (ACC3 and DEC3) underscores the importance of maintaining neuromuscular resilience across different match contexts (31, 35, 36). Given the high mechanical demands of deceleration, training programs should emphasize eccentric strength and neuromuscular conditioning to improve deceleration tolerance and mitigate fatigue-induced performance decrements (8, 36, 37).

Although this study provides important insights into MDPk and MDPm demands across multiple rolling average durations and playing positions during an elite youth international tournament, some limitations should be acknowledged. First, the kinematic and mechanical intensity thresholds were derived from studies in senior football (17, 19). While some similarities exist with thresholds used in elite youth soccer (5, 10, 18), other studies have employed different thresholds for HSR (18–21 km/h) and SPR (>21 km/h), which may contribute to discrepancies between findings. Second, although the reference team won all five matches of the tournament, the influence of match outcome and match status on MDP performance may limit the generalizability of our findings (11). Finally, integrating tactical contextualization of MDP demands across multiple durations (13, 14) may provide greater insight and improve the translation of findings into training prescription, warranting investigation in future research.

### Practical applications

This study provides critical insights into the tournament demands and the performance of the winning team. Integrating tracking and video analysis could further the understanding of the relationship between tactical and situational contexts and multifactorial MDP across different durations, thereby increasing insight into how these factors influence MDP intensities and compositions. The relationship between duration and MDP intensities and compositions highlights the importance of a comprehensive analysis that considers player characteristics, positional roles, and match-specific contexts. The inverse relationship between duration and intensity alters MDP composition, potentially reflecting shifts in energy system contributions and metabolic demands. Differential intensity profiles and MDP compositions across durations can inform player- and position-specific training strategies, ensuring that training constraints effectively target relevant physiological adaptations to enhance preparation, monitoring, and recovery. These benchmarks can inform supplemental or compensatory training drills aligned with regular training sessions, facilitating targeted locomotor activities through systematically designed constraints within sport-specific conditioning programs.

## Conclusions

In conclusion, the implemented analysis approach provides a valuable framework for evaluating peak kinematic and mechanical demands across tournaments and age groups, contributing to the improvement of tournament preparation and player development strategies. By adopting a multifactorial approach, practitioners can better understand how MDPk (MSR, HSR, SPR) and MDPm (ACC3, DEC3) contribute to overall match intensity and player workload, allowing for more precise training prescriptions that account for position-specific and contextualized kinematic and mechanical demands. Analyzing the compositional structure of MDP, rather than relying solely on absolute intensities, offers a more comprehensive understanding of individual and positional demands, enhancing player monitoring, training design, and recovery strategies. Finally, the findings of this study should be interpreted with caution, as a single team was analyzed during one tournament, which limits the sample size and the generalizability of the results.

## Data Availability

The raw data supporting the conclusions of this article will be made available by the authors, without undue reservation.
